# Inguinal ureter herniation evaluated with magnetic resonance imaging: a case report

**DOI:** 10.1186/s13256-020-02521-7

**Published:** 2020-10-27

**Authors:** Matteo Renzulli, Guido Marzocchi, Giulio Vara, Beniamino Corcioni, Anna Maria Ierardi, Caterina Gaudiano, Rita Golfieri

**Affiliations:** 1 Radiology Unit, Department of Experimental, Diagnostic and Speciality Medicine, Sant’Orsola Hospital, University of Bologna, Bologna, Italy; 2 Unit of Radiology, IRCCS Cà Granda, Ospedale Maggiore Policlinico, Milan, Italy

**Keywords:** Case reports, Emergency service, General surgery; hernia, inguinal, Magnetic resonance imaging, Ureter

## Abstract

**Background:**

The herniation of the ureter into the inguinal canal is a rare condition, but probably underreported. Acquired nephroptosis could cause herniation of the ureter and, therefore, when diagnosed, a full study of the urinary tract should be performed especially in patients with inguinal hernia.

**Case presentation:**

We present the case of an 86-year-old white man with a herniated ureter likely caused by acquired nephroptosis presenting with acute urinary retention, documented with magnetic resonance imaging for the first time.

**Conclusions:**

The Fast Imaging Employing Steady State Acquisition sequence on magnetic resonance imaging, for many reasons, could allow correct evaluation of the urinary tract, especially in cases of renal dysfunction that contraindicate the use of intravenous contrast agents.

## Background

Inguinoscrotal herniation of the ureter (native or transplanted) is an extremely rare medical condition [1], usually related to congenital abnormalities or to postoperative anatomical alterations [2]. Most (80%) ureteral inguinal hernias are paraperitoneal, meaning that an anteromedial hernial sac is present with the ureter that is drawn into the canal along the posterior aspect of the peritoneum, while some 20% of cases of ureteral inguinal hernias are extraperitoneal, meaning that a true hernial sac is not present and the ureter is accompanied by retroperitoneal fat only.

Most reported cases are noted at the time of surgical exploration for inguinal hernia repair, or later as a result of an operative injury [3].

Therefore, awareness of this anomaly is important, to avoid ureteral injury during herniorrhaphy if the anomaly is asymptomatic, and to correctly diagnose it as a possible cause for acute ipsilateral hydronephrosis in an emergency setting.

## Case presentation

An 86-year-old white man, with a pathological history of right kidney stones, presented to the emergency room of our University Hospital with right flank pain of 2 days’ duration, radiating to the ipsilateral inguinal fossa with concomitant acute urinary retention, worsening constipation, and mild dyspnea.

Our patient was affected by known bilateral massive inguinoscrotal hernias, hydrocele, and benign prostatic hyperplasia and, therefore, he was scheduled for elective hernioplasty and prostatectomy.

On physical examination, our patient presented a distended, diffusely painful abdomen with metallic peristalsis; however, the pain characteristics were not suggestive for a complication of the hernias. The known inguinoscrotal hernias were examined, they presented as a lump above and medially from the pubic tubercle, were reducible and showed no sign of incarceration. The laboratory test results demonstrated only a high level of serum creatinine and a high level of uricemia. Our patient underwent an inconclusive ultrasound due to the excess of intestinal meteorism. Therefore, a computed tomography (CT) examination was performed, without infusion of contrast media due to the low glomerular filtration rate (GFR < 28.5 mg/l). The CT scan revealed a distended bladder, severe hydronephrosis without stones in the urinary tracts, nephroptosis, and right inguinal herniation of the abdominal adipose tissue that was probably dragging on the ipsilateral ureter.

Our patient subsequently underwent urological magnetic resonance imaging (Uro-MRI), a technique with high spatial and contrast resolution, to confirm the suspected diagnosis of the right ureter herniated into the ipsilateral inguinal canal and prior to the scheduled surgical procedures of prostatectomy and hernioplasty. The MRI was carried out with the use of contrast agents, safer to administer to patients at risk of contrast-induced acute kidney injury. The Uro-MRI was performed by acquiring the standard sequences and by using the Fast Imaging Employing Steady State Acquisition (FIESTA) sequences in different spatial planes.

**Fig 1:**
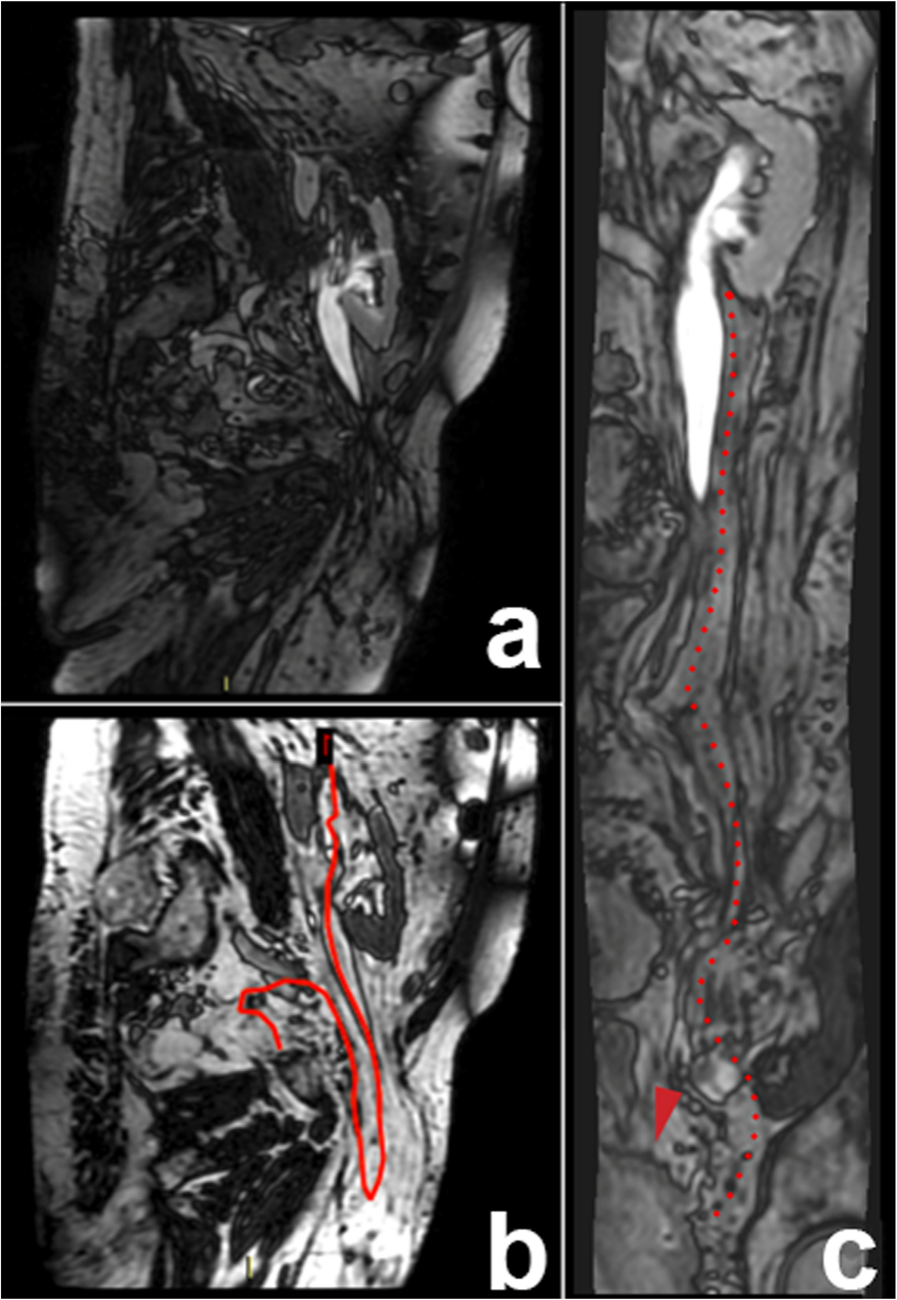
The Fast Imaging Employing Steady State Acquisition sequences show the herniated right ureter into the inguinal canal (**a**); the red line in (**b**), performed on the ureter course, from the right kidney to the bladder, was performed to obtain the curvilinear image of the entire ureter on a single plane (red dotted line) allowing the evaluation of both the lumen and the walls (**c**)

The final diagnosis was achieved through the FIESTA images, reconstructed over a curved plane so as to follow the course of the right ureter and demonstrating clearly its looping through the hernial breech into the right inguinal canal (Fig 1). The classical T1-weighted images in the urological phase confirmed the diagnosis; however, the middle tract of the herniated right ureter was not opacified differently from the proximal and distal tract. This figure was different from the FIESTA sequence. Finally, our patient received a diagnosis of inguinoscrotal herniation of his right ureter and the surgical procedures of prostatectomy and hernioplasty were anticipated; the selected approach was laparo-endoscopic repair.

## Discussion

The FIESTA sequence is widely used in the evaluation of different anatomical districts, such as vascular or biliary ones [2, 3]. Nonetheless, this sequence could be very helpful in evaluating the urinary tract for many reasons: it does not need the use of contrast media; it provides images with very short acquisition times; it uses the T2 steady state contrast mechanism providing high signal-to-noise ratio images with strong signal from fluids, while suppressing background tissue for better contrast and anatomic detail evaluation; it provides the analysis of the walls, differently from the standard T1-weighted images post-contrast media, which create “luminographic” images (without the possibility to evaluate the walls of different structures, such as biliary tract); it provides images with high spatial resolution facilitating the post processing (maximum intensity projection, volume rendering, or three-dimensional navigator techniques). In particular, in this case, the ability of the FIESTA sequences to evaluate the lumen and the walls of the ureter, allowed the evaluation of these anatomical structures even if not fluid-filled, such as the middle third herniated/strangled ureter, which was not identifiable with standard Uro-MRI sequences.

The overall incidence of the inguinal hernia in the general population is 4% with values that double in male patients over 45 years old, and with a progressively increasing incidence with age [4]. The surgical repair of the inguinal hernia represents the most frequent operation performed in surgical rooms around the world. The term “inguinal hernia” defines viscera or adipose tissue protrusions through from the abdominal cavity that contains it through the inguinal or femoral canal [5].

## Conclusions

The herniation of the ureter into the inguinal canal or scrotum is a rare but probably underreported condition in the medical literature. The possible etiology of this condition is ureter redundancy, probably due to acquired nephroptosis. Finally, the awareness of ureteral herniation into the inguinal canal is critical for the emergency physician to formulate the correct diagnosis to avoid potential ureteral damage in case of surgical repair [1, 6]. To the best of our knowledge, this case represents the first experience of inguinoscrotal herniation of the ureter documented by Uro-MRI and, in particular, evaluated by the FIESTA sequence.

## Data Availability

Data sharing not applicable to this article as no datasets were generated or analysed during the current study.
